# Supplementary material for: Analysis of PPARγ Signaling Activity in Psoriasis
Source: Int J Mol Sci. 2021 Aug 10;22(16):8603. doi: 10.3390/ijms22168603 (PMC8395241; doi:10.3390/ijms22168603)
Supplement: Supplementary file 1 [file ijms-22-08603-s001.zip › Supplemental materials_Analysis of PPARg signaling activity in psoriasis/Pathway models/Models images and html files/Differentiation of psoriatic T cells/178282.html]

inflammatory response


# Cell Process inflammatory response

|  |  |
| --- | --- |
| URN | urn:agi-gocellproc:0006954 |
| Total Entities | 1 |
| Connectivity | 10622 |
| Name | inflammatory response |
| ObjectType | Cell Process |

---

|  |  |
| --- | --- |
| ChildConcepts | kinin cascade |

---

|  |  |
| --- | --- |
| Pathway | Complement Activation in Macular Degeneration |
|  | Complement Alternative Pathway |
|  | Complement Activation by Lectin |
|  | Complement Classical Pathway |
|  | Fatty Acids Block TLR Signaling in Enteroendocrine Cell |
|  | Toll-like Receptors Act through MYD88-TIRAP Signaling |
|  | NOD-like Receptors |
|  | Toll-like Receptors Act through MYD88 Signaling |
|  | Dectin-1 (CLEC7A) Signaling |
|  | DC-SIGN (CD209) Signaling |
|  | DCIR1 (CLEC4A) Signaling |
|  | TLR4 Signaling in Leukocytes |
|  | Dectin-2 (CLEC6A)/Mincle (CLEC4E)/BDCA2 (CLEC4C) Signaling |
|  | Natural Killer Cell Activation through C-Type Lectin-like Receptors |
|  | CD8+ T-Cell Activation |
|  | Th1-Cell Differentiation |
|  | Treg-Cell Differentiation |
|  | Th17-Cell Differentiation |
|  | Plasmin Effects in Inflammation |
|  | Bradykinin Effects in Inflammation |
|  | Toll-like Receptors in Sterile Inflammation |
|  | Toll-like Receptor Independent Sterile Inflammation |
|  | Hypothalamic-Pituitary-Adrenal Axis in Acute Phase Response |
|  | Annexins in Inflammation Resolution |
|  | Vascular Endothelial Cell Activation by Blood Coagulation Factors |
|  | Dendritic Cells Function in Atherosclerosis |
|  | Th1-Cells Activation and Proliferation in Atherosclerosis |
|  | TRPM4/6/7/8 Signaling in Arterial Hypertension (Hypothesis) |
|  | Lymphocyte Mediated Myocardial Injury in Myocarditis |
|  | Atopic Dermatitis |
|  | IL17 Signaling in Psoriasis |
|  | T-Cells Differentiation Block in Psoriasis |
|  | IL22 Induces Keratinocyte Proliferation in Psoriasis |
|  | Th17-Cell Function in Systemic Lupus Erythematosus |
|  | Th1-Cell Function in Systemic Lupus Erythematosus |
|  | Complement System Defects in Systemic Lupus Erythematosis |
|  | Acute Cytotoxic CD8+T-Cell Response against Melanocytes |
|  | CD8+ T-Cell Response to Self-Determinants in Diabetes |
|  | TLR2 Signaling in Treg-Cell in Type 1 Diabetes (Animal Model) |
|  | TLR7 and TLR9 in Plasmacytoid Dendritic Cell |
|  | Immune Sustem Activation in Hashimoto's Thyroiditis |
|  | Hashimoto's Thyroiditis |
|  | Thyroid Dysfunction |
|  | Celiac Disease |
|  | Th17-Cell Activation in Crohn's Disease |
|  | Genes with Susceptibility for Crohn's Disease and Ulcerative Colitis |
|  | OipA Signaling in Helicobacter Pylori Infection |
|  | DupA Induced IL12 Production in Helicobacter Pylori Infection |
|  | Dendritic Cell Function in Ulcerative Colitis |
|  | Epithelial Cell in the Innate Immune Response in Ulcerative Colitis |
|  | T-Cell Cytotoxic Response in Ulcerative Colitis |
|  | Th17-Cell Differentiation in Asthma |
|  | CFTR Facilitates Oxidative Stress in Airway Epithelium in CF |
|  | Dendritic Cell Dysfunction in Psoriatic Arthritis |
|  | Th17-Cell and Th1 Immune Responsein Psoriatic Arthritis |
|  | Osteoclast Activation in Psoriatic Arthritis |
|  | Keratinocyte Activation in Psoriatic Arthritis |
|  | CD8+ T-Cell Action Impairment in HIV Type 1 Infection |
|  | Th17-Cell Numbers Reduction in HIV |
|  | Cathepsins Role in Periodontitis |
|  | IL1 Receptor Antagonist (IL1RN) Deficiency (DIRA) |
|  | PAPA Syndrome |
|  | TNF Receptor Associated Periodic Syndrome |
|  | Mevalonate Kinase Deficiency |
|  | Blau Syndrome |
|  | IL36 Receptor Antagonist (IL1F5) Deficiency (DITRA) |
|  | Cryopyrin (NLPR3) Associated Periodic Syndromes |
|  | Familial Cold Autoinflammatory Syndrome 2 |
|  | Familial Mediterranean Fever |
|  | Majeed Syndrome |
|  | Muscular Dystrophies |
|  | Complement Activation in Alzheimer's Disease |
|  | SOD1 Mutation |
|  | MPB-Related Complement Cascade Activation |
|  | Coagulation Factors and PAR1/2 Receptors (F2R/F2RL1) in Endometriosis |
|  | Cytokines Trigger Otitis Media |
|  | Immune System Role in Otitis Media |
|  | Plasmin Role in Glomerulonephritis |
|  | Endothelial Cell Dysfunction in Glomerulonephritis |
|  | Complement Activation in Glomerulonephritis |
|  | NFKB Canonical Signaling Activation in Cancer |
|  | Telomeric Proteins/NFKB Positive Feedback Interplay in Cancer |
|  | NF-kB Canonical Signaling |
|  | e |
|  | PPAR Psoriasis |
|  | PPARG inhibits phenotypes assotiated with psoriasis |
|  | Neighbors of inflammatory response |
|  | New Pathway (3) |
|  | Innate immunity in Leukocytes |
|  | SARS induces Complement Activation |
|  | Overview. Diseases of circulatory system (chapter 8) |
|  | Overview. Diseases of the skin (chapter 11) |
|  | Overview. Diseases of the digestive system (chapter 10) |
|  | Overview. Diseases of the musculoskeletal system (chapter 12) |
|  | Overview. Diseases of the respiratory (chapter 9) |
|  | Overview. Diseases of the genitourinary system (chapter 13) |
|  | 1\_Differentiation of psoriatic T cells |
|  | 2\_Interleukin-17 and interleukin-22 signaling in psoriasis |
|  | 1\_The role of complement in glomerulonephritis |
|  | 1\_Complement activation in age-related macular degeneration |
|  | 1\_Insufficient activation of immune response in the middle ear epithelium cells in otitis media |
|  | 2\_Lipotoxicity in NAFLD |
|  | 1\_CD4+ T cell and epithelial cell response in celiac disease |
|  | 2\_CD8+ T-cell response and mucosal damage in celiac disease |
|  | 1\_2\_Feedback relations between coagulation and fibrinolysis |
|  | Differentiation of psoriatic T cells |
|  | Interleukin-17 and interleukin-22 signaling in psoriasis |

---

|  |  |
| --- | --- |
| MedScan ID | 4130236 |

---

|  |  |
| --- | --- |
| Alias | anti-inflammatory responses |
|  | inflammatory response |
|  | anti-inflammatory response |
|  | inflammation response |
|  | inflammatory responses |
|  | inflammation responses |

---

|  |  |
| --- | --- |
| GO ID | 0006954 |

---
